# Our Contemporaries

**Published:** 1917-01

**Authors:** 


### OUR CONTEMPORARIES

The Bulletin of the Medical Society of the U. S., Vol. 1, No. 3, Dec., deals frankly with "discouraging features." It is stated that the Southern Clinic is the only independent journal which has actively supported the new movement. We quote the following paragraph in full:

"If other so-called 'independent' journals would be equally outspoken in support of our new organization we would have

50,000 members in 1917—which we may have, even in the face of their non-support. It is a little strange that journals which have been so full of condemnation of the methods of the present managers of the A. M. A. have remained silent about our new society. Perhaps they are waiting to see what it is going to amount to before espousing its cause. So few of us care to be associated with failures. Possibly (let us hope so), they feel that the best way to effect a reform of the mistakes of the A. M. A. is from within that body rather than through organization of a new society; just as friend Teddy of the Progressives thought of the Republican party; if so, let us see 'em "get a move on." Meantime our Progressive Party in Medicine will go straight ahead until we attain sufficient strength to say to the older society: Your vicious ways must be changed! and we shall have the necessary force of numbers to **compel** reform in the rival organization."

We think that the plain fact of the matter is that the independent journals agree with the opinion expressed by this journal in commenting (by request) on the proposition to establish this society. They feel that there should be one national organization, and that whatever evils exist in the A. M. A. should be corrected from within. As an editor and as an individual member of the A. M. A., we have expressed the opinion that certain changes should be made in that body, mainly in the way of more elastic and more popular methods of control. Our pages have never "been full of condemnations of the present managers" and, as a general rule, we do not characterize as vicious an opinion or method that differs from our conceptions of what is best. This new society should also realize that it has raised certain issues which require very careful judgment by the physician contemplating membership. It is easy to say that the code of ethics is antiquated and requires revision, and that it should be interpreted according to its spirit and not its letter. We fully agree with the principle that fees for medical and surgical attendance should be equable and proportionate to skill and labor, rather than to kind of labor performed, and that neither should have a prior claim. We go so far as to say that there are many instances of joint attendance in which the best way to solve the fee-splitting problem is to take the bull by the horns and to split the common bill, equably, even equally—but to do so honestly and openly. If, as charged, fellows of the A. M. A. and of the College of Surgeons have got around the provisions against dishonorable fee-splitting by paying assistants (up to $18,000 a year in one instance) who were not really assistants but touts, the proper course is evident. But the new society must make it plain that its revisions of the code of ethics are ethical and sincere.

---

The Medical Review of Reviews announces a symposium on the Medical Profession from the Standpoint of the Laity, including the names of Andrew Carnegie, John Wanamaker, Nathan Strauss, Israel Zangwill, W. D. Howells, Geo. W. Cable, John Kendrick Bangs, John Philip Sousa, David Starr Jordan, Booker T. Washington, Nikola Tesla and Hudson Maxim and others.

---

The Louisville Journal of Medicine and Surgery will, with the Jan. issue, become the Mississippi Valley Medical Journal the official organ of the corresponding Association.

Dr. Henry Enos Tuley, Secretary of the Association, will continue as Editor, and a special Editorial Committee, composed of the following, will assist in the editorial policy of the Journal: Drs. William N. Wishard, Indianapolis; Arthur R. Elliott, Chicago; Willard J. Stone, Toledo, Ohio, and Louis Frank, Louisville. A special epitome department will be established, giving each month a review of some special border line topic which is of interest to surgeon and internist.

